# Rates of central line associated bloodstream infection in tertiary care hospitals in three Arabian Gulf countries; six-year surveillance study

**DOI:** 10.1186/2047-2994-4-S1-O22

**Published:** 2015-06-16

**Authors:** HH Balkhy, A El-Saed, H Alansari, A Althaqafi, J Jameela, Z AL Maskari, A El Gammal, W Al Nasser, A AlJardani, SS Al-Abri

**Affiliations:** 1 Infection control, King Abdulaziz Medical City, Riyadh, Saudi Arabia; 2 Infection control, Salmaniya Medical Complex, Manama, Bahrain; 3 Infection control, King Abdulaziz Medical City, Jeddah, Saudi Arabia; 4 Infection control, Royal Hospital, Muscat, Oman; 5 Infection control, King Abdulaziz Hospital, Al Hassa, Saudi Arabia; 6 Infection control, Imam Abdulrahman bin Faisal Hospital, Dammam, Saudi Arabia

## Introduction

There is lack of benchmarking data for central line associated bloodstream infection (CLABSI) in Gulf Cooperation Council (GCC) countries. Available data are small isolated reports that do not cover different hospital locations.

## Objectives

To estimate location-specific CLABSI rates in GCC region and to compare such rates with published reports of US National Healthcare Safety Network (NHSN) and International Nosocomial Infection Control Consortium (INICC).

## Methods

CLABSI rates and central line utilization between 2008 and 2013 were calculated from aggregated CLABSI surveillance data using NHSN methodology pooled from different ICUs and oncology wards in 6 hospitals in three GCC countries; Saudi Arabia, Oman, and Bahrain. Standardized infection ratio (SIR) of CLABSI in GCC hospitals were compared with published reports of NHSN and INICC.

## Results

A total 461 CLABSI events were detected during 6 years of surveillance covering 150,492 central line-days and 336,850 patient-days. The overall CLABSI rate was 3.1 per 1000 central line-days (95% CI 2.8-3.3) and overall central line utilization was 0.45. The CLABSI rates showed 40% reduction while central line utilization showed 12% reduction towards the end of surveillance period. CLABSI rates were highest in neonatal ICUs (5.0) and adult oncology wards (4.8) while central line utilization were highest in pediatric (0.86) and adult cardiothoracic ICUs (0.80) and pediatric oncology wards (0.76). After adjusting for differences in ICU types, the risk of CLABSI in GCC hospitals was 146% higher than NHSN hospitals but 33% lower than INICC hospitals. Similar to NHSN hospitals, the majority of CLABSI events (81%) were diagnosed by detecting recognized pathogens in blood cultures.

## Conclusion

The risk of CLABSI in ICU and oncology patients in GCC countries is probably much higher than the US but the risk in ICU patients is slightly lower than many developing countries. Current findings may be used as a regional benchmark.

## Disclosure of interest

None declared.

